# Genes and Podocytes – New Insights into Mechanisms of Podocytopathy

**DOI:** 10.3389/fendo.2014.00226

**Published:** 2015-01-23

**Authors:** Agnieszka Bierzynska, Katrina Soderquest, Ania Koziell

**Affiliations:** ^1^Academic Renal Unit, School of Clinical Sciences, Bristol University, Bristol, UK; ^2^Division of Transplantation Immunology and Mucosal Biology, Department of Experimental Immunobiology, Faculty of Life Sciences and Medicine, King’s College London, London, UK

**Keywords:** podocyte nephropathy, podocytes, genetic predisposition to disease, gene mutation, nephrotic syndrome, nephrotic genes

## Abstract

After decades of primarily morphological study, positional cloning of the *NPHS1* gene was the landmark event that established aberrant podocyte genetics as a pivotal cause of malfunction of the glomerular filter. This ended any uncertainty whether genetic mutation plays a significant role in hereditary nephrotic syndromes (NS) and confirmed podocytes as critical players in regulating glomerular protein filtration. Although subsequent sequencing of candidate genes chosen on the basis of podocyte biology had less success, unbiased analysis of genetically informative kindreds and syndromic disease has led to further gene discovery. However, the 45 genes currently associated with human NS explain not more than 20–30% of hereditary and only 10–20% of sporadic cases. It is becoming increasingly clear both from genetic analysis and phenotypic data – including occasional response to immunosuppressive agents and post-transplant disease recurrence in Mendelian disease – that monogenic inheritance of abnormalities in podocyte-specific genes disrupting filter function is only part of the story. Recent advances in genetic screening technology combined with increasingly robust bioinformatics are set to allow identification and characterization of novel disease causing variants and more importantly, disease modifying genes. Emerging data also support a significant but incompletely characterized immunoregulatory component.

## Nephrotic Syndrome and the Glomerular Filtration Barrier

Nephrotic syndrome (NS) is one of the commonest kidney conditions to affect children and adults. It manifests as excessive leak of protein into the urine, with interstitial edema occurring through albumin loss, aggravated by salt and water retention. Moreover, secondary knock-on effects on lipid metabolism, hemostasis, and the endocrine system through loss of key binding proteins serve to augment morbidity and mortality. NS represents a heterogeneous group of conditions, some idiopathic, others with a clear genetic basis. It may be highly kidney specific, associated with extra-renal developmental malformations or occur as a complication of systemic disease. The mainstay of treatment is immunosuppression, also effective in about 8–10% of genetic cases. Non-responders generally progress to renal failure with kidney transplantation, the only life-saving treatment.

## The Role of Podocytes

Despite the apparent heterogeneity, the unifying feature in NS is malfunction of the glomerular filtration barrier (GFB). This highly sophisticated macromolecular sieve with size and charge restricting characteristics is the primary ultra-filter of plasma by the kidney. It allows free flow of water and small solutes but restricts the passage of molecules >15 kDa; proteinuria occurs through loss of these normal permselective properties. The GFB comprises three layers: fenestrated endothelium, glomerular basement membrane (GBM) and podocytes, specialized terminally differentiated epithelial cells connected by slit diaphragms (SD), unique intercellular junctions interposed between interdigitating foot processes (1). Endothelial cells and podocytes have a negatively charged surface glycocalyx, which together with GBM sialoproteins and heparan sulfate gives the GFB an overall negative charge at physiological pH. Although size is the primary determinant of molecular filterability, recent detection of mutations in the main component of the podocyte glycocalyx, podocalyxin, in familial NS (2) supports additional charge selection through electrostatic repulsion.

Although damage to any of the three layers can result in significant proteinuria and kidney disease (3), podocytes are considered pivotal for maintaining barrier integrity. They encircle the glomerular capillaries creating a compact interdigitating network on the urinary side of the GFB. The connecting SDs integrate structural components of tight, adhesion, gap, and neuronal junctions to meet the diverse functional requirements of macromolecular filtering under high pressure while subjected to rapid changes in mechanical shear stress (4). SDs connect with the actin cytoskeleton to initiate signaling pathways that regulate podocyte function, namely plasticity of foot processes, mechanosensation, calcium flux, endocytosis, cell polarity, and cell survival. It is perhaps not surprising that podocyte gene mutations link to these key cellular functions. Podocytes also react very stereotypically to injury irrespective of whether this is acquired or resulting from an intrinsic developmental defect with reorganization of the actin cytoskeleton, foot process effacement, molecular re-characterization of SDs, apoptosis, and detachment from the GBM (5). These dramatic morphological changes correlate with dysregulation of specific markers of podocyte differentiation including WT1, PAX2, and nephrin again signifying an underlying molecular basis. Additionally, de-differentiated podocytes can attempt healing sometimes by excessive proliferation, with the eventual outcome of repair or cell death attributable to complex interplay of poorly defined genetic and epigenetic mechanisms (6–8).

Clinical data support the hypothesis that the predominant cellular lesion targets podocytes; GBM gene defects usually result in insidious plasma protein leak whereas defects in podocyte/slit-diaphragm genes cause precipitous leak and NS. Moreover, there is evidence for a potential role for immune-regulation, particularly in childhood. Podocytes express cytokine and chemokine receptors as well as Toll Like receptors (TLRs) (9) and can respond to immune stimuli both *in vivo* and *in vitro*. Moreover, emerging evidence indicates that podocyte injury in NS may sometimes result from an unknown circulating factor, cytokine imbalance, or immune complex injury, with rare genomic variants potentially dictating susceptibility or resistance to immune triggers and the degree of subsequent response.

## Advances in Molecular Characterization of Podocytes

Initial evidence supporting a molecular basis for NS came from positional cloning in familial cases and experimental animal models. Inherited diseases are frequently caused by mutations in genes with restricted expression patterns that generally do not cause early embryonic lethality but instead manifest at the time when gene function becomes critical for a specific tissue and subsequent survival (10). This is certainly the case for *NPHS1* and *NPHS2* (11, 12), the first podocyte genes cloned in NS encoding nephrin, an immunoglobulin superfamily member, and podocin an integral membrane protein both exclusively expressed at the podocyte SD supporting a key role in protein filtration. Mutations are associated with autosomal recessive NS manifesting at birth or early life during post-natal glomerular maturation and resultant increase in glomerular filtration.

Detection of *WT1* mutations in human syndromic NS (13), together with murine transgenic approaches using *WT1, CD2AP* (14), and *NEPH1* (15) underscored the contribution of developmentally regulated podocyte genes. Subsequent identification of mutations in *ACTN4* (16) and *INF2* (17) emphasized the central role of the actin cytoskeleton whereas gain of function mutations in *TRCP6* linked podocyte disease to abnormalities of cellular calcium flux and associated signaling pathways (18). Inherited defects of mitochondrial (19) and lysosomal components (20) have also been shown to lead to profound podocyte dysfunction, suggesting that they have high energy requirements and turn over. This also lead to the discovery that lysosomes participate in autophagy, increasingly recognized as protecting podocytes against injury (21, 22) Other genes such as *CD2AP* that participate in endocytosis and/or actin assembly have also been detected in glomerular disease providing further important clues about podocyte biology.

Familial and candidate gene studies have provided important information about the molecular basis of fundamental podocyte functions such as slit-diaphragm signaling, regulation of actin cytoskeleton dynamics, and cell–matrix interactions. However, until recently, only limited information about the overall genetic landscape was available, which posed a challenge to correct interpretation of genetic findings. Previously, podocytes could be extracted with high purity, but low cell yields during isolation hampered complete characterization of the transcriptome and proteome. Primary cells are difficult to grow in culture, so immortalized podocytes provided a useful tool to study gene function but incompletely mirrored their *in vivo* counterparts at molecular level. However, recent development of a double fluorescent reporter mouse model and optimized bead perfusion protocol, combined with FACS sorting, microarray, proteomics, and unbiased mathematical biology has yielded >5223 differentially regulated genes within the podocyte transcriptome and >1280 translated proteins (23). These studies have revealed that the podocyte proteome is enriched with plasma membrane, cytoskeleton, and neuronal-type proteins. Subsequent functional analysis of transcriptome and proteome data resulted in seven gene clusters: endoplasmatic reticulum, ubiquitination, cytoskeleton, nuclear elements, mitochondria, peroxisome and protein transport, and cell junction. Interestingly, nuclear and translational proteins were depleted, reflecting the post-mitotic nature of podocytes and alternative splicing was reduced, perhaps to ensure discreet cellular regulation. This has also enabled more meaningful RNA sequencing, which has identified eight miRNA’s preferentially expressed in podocytes with significantly reduction fold change of miRNA target genes supporting miRNA-mediated mRNA degradation.

Improved description of the podocyte genome has also resulted in more effective interpretation of data from next generation sequencing (NGS) and to detect new variants or genes that cause or contribute to NS. NGS allows rapid sequencing of the whole exome or genome, and is becoming an increasingly powerful and cost effective method of analyzing the genetic contribution to any disorder. An unbiased approach can be taken to highlight new genes, new pathways, and new disease mechanisms through rigorous statistical genetics followed by functional experiments. This is especially relevant to NS where there is already known association with complex genetic determinants and this is likely to prove important mechanistically. However, as with any rare diseases, finding causal variants in NS suffers from twin problems of moderately low sample size and disease heterogeneity. In presumed Mendelian NS, the approach is still to search for genetic variants that segregate with disease within a family. In sporadic NS, we hypothesize that the condition will be caused by a rare variant in a small number of cases (limiting sample size and statistical power) and that the variant and, indeed the gene, may vary between cases. Added to this, although it is often assumed that there will be a heterozygous, homozygous, or compound heterozygous change that is causal, in common with related glomerular disorders, there is evidence for modifier effects in NS, at the genetic (24) and environmental level (25). This introduces the concept that NS is more complex than the Mendelian one podocyte gene, one disease situation as previously thought as variants are not always completely penetrant (26), synergistic activation between genes may occur and in non-heritable disease, an environmental trigger is needed to cause disease. There is now clear evidence that in at least one podocyte gene, the pathogenicity of a particular allele depends on the trans-associated mutation (27). These concepts are supported by increasing evidence that expected phenotype–genotype correlations are not always adhered to and pathological changes may develop focally (not in all glomeruli) and segmentally (only in parts of a glomerulus), a pattern suggestive of an initial insult precipitated by an environmental stressor, perhaps a viral infection, that leads to localized cell injury (28).

Attempts to analyze NGS data in rare yet moderately complex diseases such as NS initially tend to focus on three main issues, namely how to best divide the genome for maximum signal, which statistical test to use and how to computationally assess the function of any variants found. Unlike common complex conditions, variants in conditions such as NS are too rare to provide meaningful sample sizes for statistical testing on their own and must be collapsed or aggregated to the level of gene, network, or other biologically meaningful unit. That is, testing for enrichment of variants in NS versus a control population will likely involve counting the number of rare protein altering variants in each gene or other biological unit in cases and controls and finding genes with an excess of these rare variants, rather than searching for an enrichment of any one variant as would be the case in a common complex Genome Wide Association Study (GWAS) approach. In addition to deciding on the biological unit by which to combine variants, decisions also need to be made about the statistical techniques used to combine and then analyze variants (29, 30). There are then several databases and algorithms by which the functional deleteriousness of potential variants can be assessed [(31): CADD; (32): ClinVar; (33): HGMD; (34): SIFT; (35): PolyPhen]. These might be used throughout the analysis to remove samples with known causal variants before statistical testing, or choose likely deleterious variants during testing. They can also be used to examine the likelihood that new variants might be causal prior to investing resources in replication studies or functional testing *in vitro* or *in vivo*. Nonetheless, understanding genotype/phenotype relationships without powerful systems biology tools in place has become increasingly complicated as emerging NGS data reveal not only considerable genetic heterogeneity but also evidence showing that factors such as epigenetic modifications, imprinting, non-coding RNAs, and RNA editing may play an important role in determining phenotype (36).

## Podocyte-Specific Genes: The Old and The New

**Figure 1:**
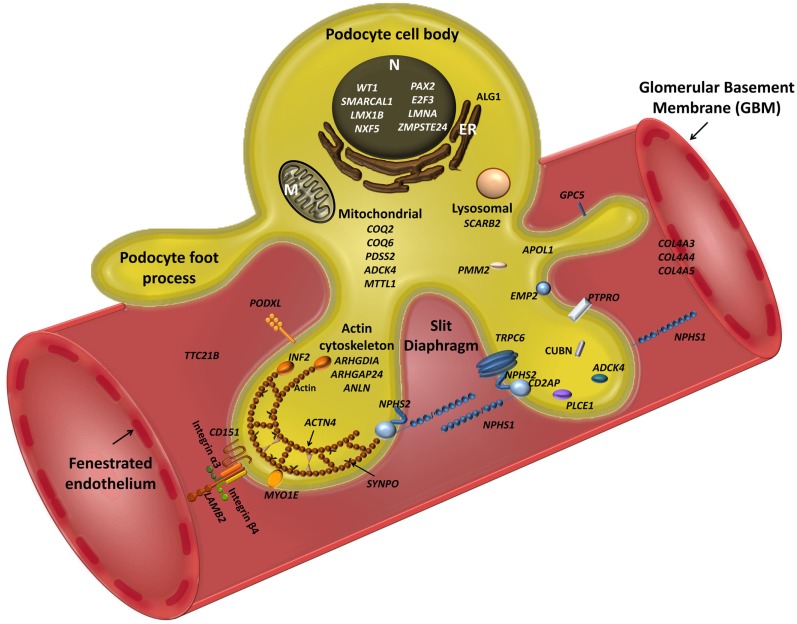
**Schematic view of podocyte genes associated with steroid resistant nephrotic syndrome (with reference to Table 1)**. Podocyte cell with foot processes, slit diaphragm, the glomerular basement membrane (GBM), and fenestrated endothelium are shown. *N*, podocyte nucleus; ER, endoplasmic reticulum; M, mitochondrion. Slit diaphragm associated and adaptor proteins: nephrin (*NPHS1*), podocin (*NPHS2*), CD2-associated protein (*CD2AP*), PLCε1 (*PLCE1*), transient receptor potential channel 6 (*TRPC6*), and protein tyrosine phosphatase receptor type O (*PTPRO*). Nuclear proteins and transcription factors: Wilm’s tumor protein (*WT1*), SWI/SNF-related matrix-associated actin-dependent regulator of chromatin subfamily A-like protein 1 (*SMARCAL1*), LIM Homeobox Transcription Factor 1β (*LMX1B*), nuclear RNA export factor 5 (*NXF5*), paired box protein (*PAX2*) and transcription factor E2F3 (*E2F3*), lamin A/C (*LMNA*), zinc metallopeptidase STE24 (*ZMPSTE24*). Actin cytoskeleton and signalling: podocalyxin (*PODXL*), inverted formin 2 (*INF2*), α-actinin-4 (*ACTN4*), synaptopodin (*SYNPO*), myosin 1E (*MYO1E*), Rho-GDP-dissociation inhibitor 1 (*ARHGDIA*), and Rho-GTPase-activating protein 24 (*ARHGAP24*), and actin-binding protein anillin (*ANLN*), cubilin (*CUBN*), tetratricopeptide repeat protein 21B (IFT139, *TTC21B*); epithelial membrane protein 2 (*EMP2*). Mitochondria-associated proteins: parahydroxybenzoate-polyprenyl transferase (*COQ2*), ubiquinone biosynthesis monooxygenase (*COQ6*), decaprenyl-diphosphate synthase subunit 2 (*PDSS2*), AarF domain-containing kinase 4 (*ADCK4*), and mitochondrially encoded tRNA leucine 1 (*MTTL1*). Metabolic and lysosomal proteins: chitobiosyldiphosphodolichol β-mannosyltransferase (*ALG1*), phosphomannomutase 2 (*PMM2*), and scavenger receptor class B, member 2 (*SCARB2*). GBM associated proteins: collagen 4 – *COL4A3, 4, 5*; integrin-α3 (*ITGA3*), integrin-β4 (*ITGB4*), laminin-β2 (*LAMB2*); CD151 antigen (*CD151*).

Mutations in 45 genes have been associated with familial and sporadic NS to date (Figure 1). These disrupt function either through SD disassembly, damaging cell architecture or metabolism, disturbing cell–matrix interactions, and/or impeding signaling pathways. All are expressed in podocytes, but there is increasing evidence to suggest that other systems, including immunoregulatory, play a role. Moreover, mutations in podocyte genes currently only explain 20–30% of familial and 10–20% of sporadic NS. From a clinical perspective, the renal phenotype rarely correlates absolutely with genotype as histology overlaps, suggesting a final common pathway to glomerular damage. Nonetheless, mode of inheritance and age of onset can give important clues. Gene mutations detected in early life are biased toward developmental genes, podocyte/SD/GBM malformation, and autosomal recessive inheritance whereas in later life, mutations are more frequently autosomal dominant (AD), preferentially affecting genes that participate directly or indirectly in regulation of the actin cytoskeleton. Moreover, combined gene defects in more than one podocyte gene may play a role in the development of NS, for example, mutations in both *NPHS1* and *NPHS2* can cause a tri-allelic hit modifying phenotype (37), and bi-allelic trans-heterozygosity has been described for *CD2AP* and *NPHS2* in sporadic NS (38). Additionally, *ADCK4* appears to modify CoQ10 (39) Furthermore, R229Q, a non-neutral *NPHS2* polymorphism that may predispose to NS in adults appears pathogenic only when associated with 3′ *NPHS2* mutations in trans- (27), or deleterious mutation in another podocyte gene (37). Another emerging layer of complexity is miRNAs, for example, miR-193a downregulates the expression of WT1 in transgenic mice resulting in rapid and progressive NS (40). Other genes regulate podocyte differentiation and SD function by interacting synergistically on common enhancers or repressors, e.g., LMX1B, which combinatorially regulates *NPHS2* with FoxC (41). This underlines the likely diversity of genetic interaction within podocytes, supporting a requirement for additional environmental factors and/or modifier genes for full phenotypic expression.

**Table 1:** **Genes directly associated nephrotic syndrome**.

Gene	Inheritance	Disease association and onset
**SD ASSOCIATED AND ADAPTOR PROTEINS**		
*NPHS1*	AR	CNS/NS
*NPHS2*	AR	CNS, NS – childhood and adult onset
*CD2AP*	?	Early-onset NS, HIV nephropathy
*PLCe1*	AR	Early-onset NS
*TRPC6*	AD	Adult onset NS
*PTPRO*	AR	Childhood-onset NS

**NUCLEAR PROTEINS**		
*WT1*	Sporadic; AD	Adult onset NS, Denys–Drash and Frasier Syndromes
*LMX1B*	AR	Nail–Patella Syndrome/NS only
*SMARCAL1*	AR	Schimke immuno-osseous dysplasia
*E2F3*	Chromosomal deletion	Early-onset NS and mental retardation
*NXF5*	X-linked recessive	NS with co-segregating heart block disorder
*PAX2*	AD	Adult onset NS
*WDR73*	AR	Galloway–Mowat Syndrome

**ACTIN CYTOSKELETON AND SIGNALING**		
*ACTN4*	AD	Adult onset NS
*MYH9*	Risk allele	Adult onset NS
*INF2*	AD	Familial/sporadic NS; Charcot-Marie-Tooth
*SYNPO*	?	Adult onset NS
*APOL1*	Complex; AR	Adult onset NS
*MYO1E*	AR	Early or adult onset NS
*ARHGAP24*	AD	Adult onset NS
*ARHGDIA*	AR	CNS
*ANLN*	AD	Adult onset NS
*EMP2*	AR	Childhood-onset NS
*CUBN*	AR	Intermittent nephrotic range proteinuria and epilepsy
*GPC5*	Risk allele	Adult onset NS
*PODXL*	AD	Early or adult onset NS
*TTC21B*	AR	NS with tubulointerstitial involvement
*CLTA4*	Risk allele	Sporadic NS

**MITOCHONDRIAL**		
*MTTL1*	?	MELAS syndrome; NS ± deafness and diabetes
*tRNAIle*	?	Deafness, NS, epilepsy, and dilated cardiomyopathy
*tRNAAsn*	?	Multiorgan failure and NS
*tRNATyr*	?	Mitochondrial cytopathy and NS
*COQ2*	AR	Mitochondrial disease/isolated nephropathy
*COQ6*	AR	NS with sensorineural deafness
*ZMPSTE24*	AR	Mandibuloacral dysplasia with NS
*PDSS2*	AR	Leigh syndrome
*ADCK4*	AR	NS
*CYP11B2*	Risk allele	C-344T SNP risk factor for IgA nephropathy, NS, proliferative glomerulopathy

**GBM**		
*LAMB2*	AR	Pierson syndrome: CNS with ocular abnormalities; isolated early-onset NS
*ITGB4*	AR	NEP syndrome-NS, epidermolysis bullosa, and pulmonary disease
*ITGA3*	AR	Epidermolysis bullosa and pyloric atresia + NS
*LMNA*	AD	Familial partial lipodystrophy + NS
*COL4A3*	AR	Alport’s disease
*COL4A4*	AR	Alport’s disease
*COL4A5*	X-linked	Alport’s disease
*CD151*	AR	NS, pretibial bullous skin lesions, neurosensory deafness, bilateral lacrimal duct stenosis, nail dystrophy, thalassemia minor

**OTHER – METABOLIC OR LYSOSOMAL**		
*PMM2*	AR	Congenital disorder of glycosylation
*ALG1*	AR	Congenital disorder of glycosylation
*SCARB2* (lysosome)	AR	Action myoclonus renal failure syndrome ± hearing loss

*AR, autosomal recessive; AD, autosomal dominant; CNS, congenital nephrotic syndrome; NS, nephrotic syndrome; ?, inheritance neither clearly autosomal dominant or recessive, possibly complex*.

Table 1 summarizes all genes currently associated with human NS. Broadly speaking, these can be categorized on the basis of function substantiated by the predicted effect of mutations. SD associated and adaptor proteins normally communicate between the SD and the podocyte cytoskeleton, forming multimeric signaling complexes associated with lipid raft micro-domains coordinating actin remodeling, cell survival, and endocytosis. The prototypes are nephrin (*NPHS1*), and podocin (*NPHS2)*, which interact with proteins such as CD2AP (42) and TRPC6 to adapt between SD and cytoskeleton through a number of cytoskeletal linkers including F-actin and synaptopodin (43). More than 236 mutations in *NPHS1* have been described (HGMD^®^ Professional 2014.2), and 171 mutations in *NPHS2* (http://www.lovd.nl/NPHS2, HGMD^®^ Professional 2014.2). *CD2AP* haploinsufficiency causes NS, and is associated with trans-heterozygosity (38). Gain of function TRPC6 mutations cause AD NS mainly but not exclusively in adult life, by increasing calcium influx and dysregulating the actin cytoskeleton via aberrant signaling (18). Nuclear proteins regulate transcription, podocyte differentiation and homeostasis; mutations result in dysregulation of downstream targets often initiating catastrophic collapse of the entire podocyte-stabilizing system. *WT1* mutations cause Denys–Drash (intersex, Wilm’s tumor, NS) and Frasier syndromes (intersex, NS, gonadoblastoma) and AD adult onset NS (44). *LMX1B*, a homeobox transcription factor, regulates SDs and is essential for the maintenance of the actin cytoskeleton. Haploinsufficiency causes Nail–Patella syndrome, a rare AD NS that can occur as just kidney specific disease (45). The most recent gene to be cloned is *WDR73*, its exact function in the podocyte is as yet unknown but mutations result in Galloway–Mowat syndrome, a rare association between microcephaly, diaphragmatic hernia, and SRNS (46). Proteins regulating the actin cytoskeleton are also targeted. *ACTN4* (α-actinin-4) causes AD adult onset NS with incomplete penetrance. Here, mutant proteins show stronger affinity for F-actin and impair cytoskeletal function. Another actin regulating gene, *INF2* belongs to the formin family and normally homodimerizes to inhibit actin depolymerization. Mutations are detected in a significant proportion of familial but not sporadic NS. Mutations in *PLCe1* (phospholipase C epsilon) mostly cause early-onset NS by dysregulating the cytoskeleton and signaling, and may be variably penetrant with some missense mutations resulting in later onset disease responding to immunosuppression (47). *PLCe1* mutations may also co-exist with trans-heterozygote mutations in other podocyte genes. *PTPRO* encodes a protein tyrosine phosphatase receptor located on the apical side of podocyte foot processes that controls the glomerular pressure/filtration rate (48). Only two splice-site mutations have been detected in *PTPRO* and cases partially responded to immunosuppression (49). Recently, mutations in *ANLN* (anillin), another F-Actin binding protein, were detected in familial NS (50), and other mutation screens of inherited disease have identified *ARHGDIA* (51) and *ARHGAP24* (52), underpinning a key role for RHO GTPases and Rho–Rac signaling in regulating the podocyte cytoskeleton. *MYO1E* (53) and *MYH9* (24), mutated in autosomal recessive NS regulate actin cytoskeletal function and cell shape. *APOL1* mutations also result in increased NS in African Americans but the mechanism remains unclear (54). Mutations affecting protein expressed in the GBM may also disrupt GFB functions sufficiently to cause NS. *LAMB2* mutations, encoding laminin β2, cause Pierson’s syndrome characterized by early-onset NS with or without microcoria, depending on the severity of the mutation (55). *ITGA3* and *ITGB4* encode integrins thought to be mainly passive GBM stabilizers, with mutations causing hyperglycosylation that prevents formation of functional integrins manifesting as NS and skin defects (56). COL4A3, A4, and A5 are key GBM proteins that cause Alport’s syndrome; recent data suggest caution in interpreting COL4A variants detected in apparent familial NS (57). Mitochondrial and other rare metabolic syndromes also present with NS, by disrupting podocyte metabolism.

Initial identification of monogenic defects correlating with morphological findings in podocytes in health and disease provided important mechanistic insights. However, this is now too simplistic, a model; podocyte genetic mechanisms are becoming increasingly complex, perhaps not surprising considering their super-specialist role within the GFB. Multiple levels of control involving modifier genes and di-genic and multi-genic inheritance are undoubtedly necessary to deal with epigenetic events and environmental effects (58). It is also likely that some gene variants are not directly causative, but rather modifiers of phenotype that may even arise from primary immune injury or presence of a circulating permeability factor. The advent of NGS and increasingly sophisticated bioinformatics approaches pave the way for exciting exploration of these alternative mechanisms, which undoubtedly play a key role in podocyte pathobiology.

## Conflict of Interest Statement

The authors declare that the research was conducted in the absence of any commercial or financial relationships that could be construed as a potential conflict of interest.
